# Supplementary figures and images for: The Impact of Age on Statin-Related Glycemia: A Propensity Score-Matched Cohort Study in Korea
Source: Healthcare (Basel). 2022 Apr 22;10(5):777. doi: 10.3390/healthcare10050777 (PMC9141400; doi:10.3390/healthcare10050777)

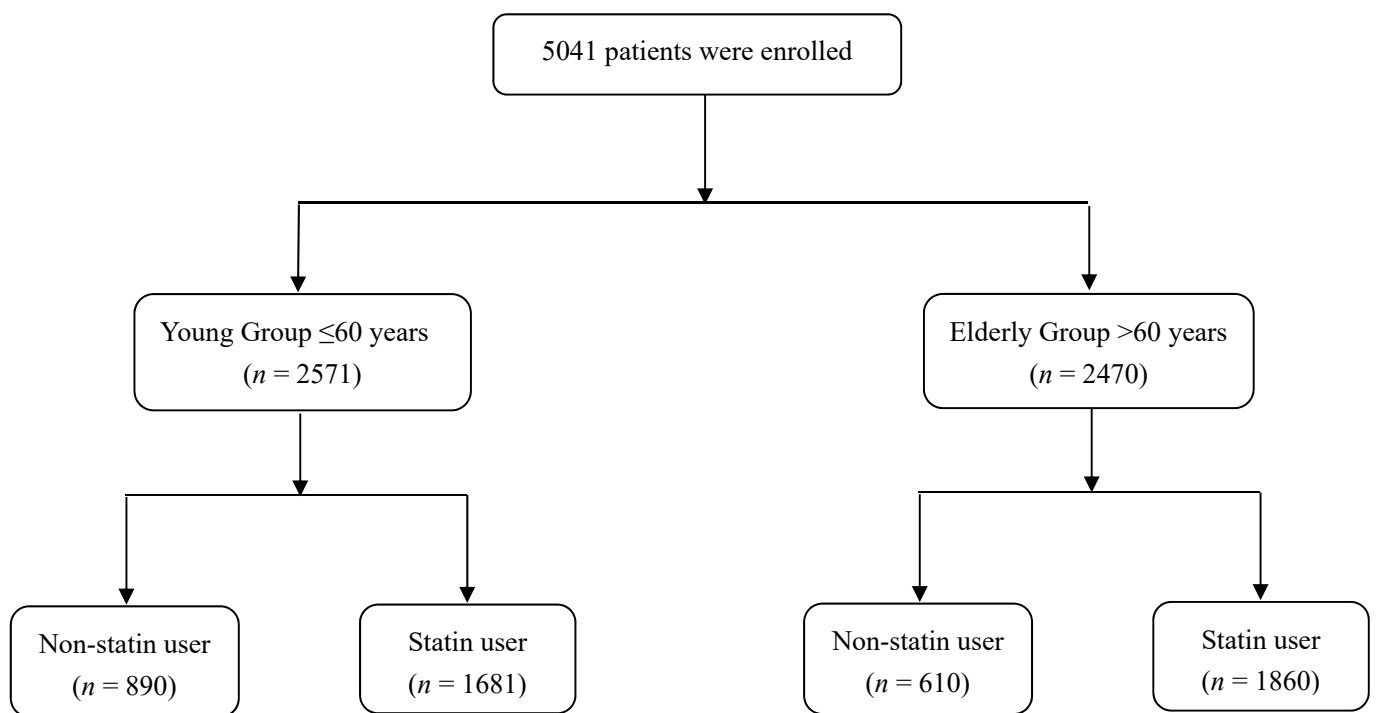

**Supplemental Figure S1.** Study participants recruitment flowchart.

Supplement: Supplementary file 1 [file healthcare-10-00777-s001.zip › healthcare-1457606-supplementary.pdf]
